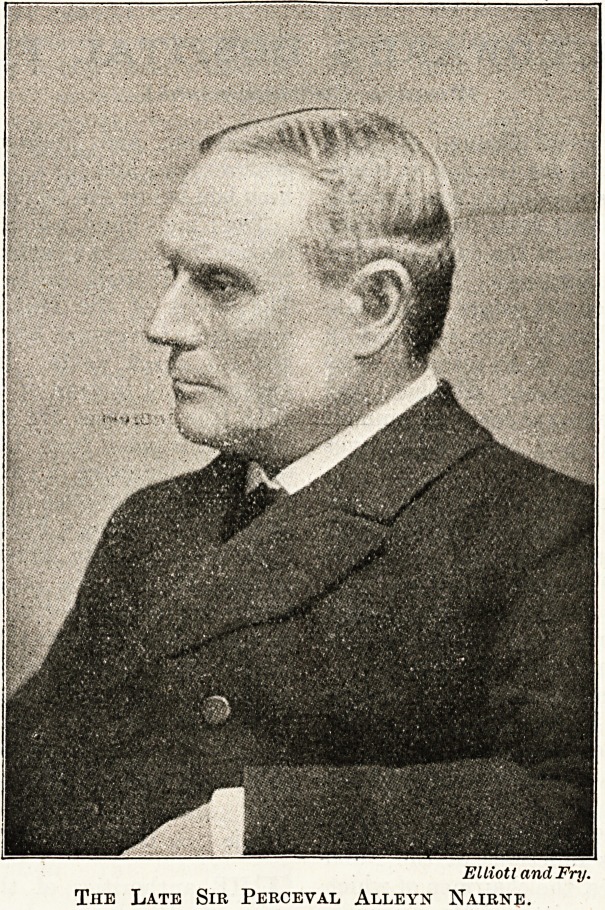# The Late Sir Perceval A. Nairne

**Published:** 1922-02

**Authors:** 


### THE OLDEST HOSPITAL CHAIRMAN.

### SIR PERCEVAL ALLEYN NAIRNE.

SIR PERCEVAL ALLEYN NAIRNE, Chairman of the Seamen's Hospital Society, died suddenly at his residence, 176 The Grove, Camberwell, on Saturday, December 10, within two months of his eighty-first birthday.

Sir Perceval was the fifth son of Captain Alexander Nairne, H.E.I.C.S., who himself was a member of the Committee of Management of the Seamen's Hospital Society from the year 1836 to 1866, and was one of Nelson's officers at the Battles of Copenhagen and Trafalgar. Admiral William Young, Sir Perceval's great-uncle, was one of the Governors appointed by the Act of Parliament incorporating the Society in 1833.

*Elliott and Fry.*

THE LATE SIR PERCEVAL ALLEYN NAIRNE.

The early years of Sir Perceval were spent at Clapham Grammar School. He was admitted a solicitor in 1864 and was the senior partner in the firm of Messrs. Baker & Nairne, of 3 Crosby Square, Bishopsgate. He was actively engaged in his professional duties till within a few days of his death.

Sir Perceval took a deep interest in matters connected with education and became a School Manager at the early age of eighteen. He was a Governor of Wilson's Grammar School and the Mary Datchelor School, Camberwell, and had occupied the position of Manager of two groups of schools under the London County Council. He was appointed General Solicitor to the National Union of Teachers, which office he held for over thirty years.

But the work which perhaps claimed his deepest sympathy was that of caring for sick and injured sailors—the work in which his ancestors had played so conspicuous a part. Sir Perceval was elected a member of the Committee of Management of the Seamen's Hospital Society (Dreadnought) on February 3, 1869, taking his seat for the first time on February 19 of that year. This Society has therefore had the advantage of being guided by his wise counsel and clear judgment for over half a century, and it is not too much to say that the Corporation owes mainly to him the success which has attended its efforts during this period of its existence.

In 1886 Sir Perceval Nairne was elected Deputy Chairman of the Seamen's Hospital Society, and on the death of Admiral the Hon. Francis Egerton in 1898 the Committee elected him their Chairman.

The period during which Sir Perceval was associated with the Dreadnought was one of remarkable progress. In 1869, when he joined the Committee, the Society's establishment consisted of an old battleship—the "Dreadnought"—lying off Greenwich. In the following year the patients were removed to the present building on shore with 250 beds. The Society now consists of the following additional establishments—the Albert Dock Hospital with 50 beds; the Hospital for Tropical Diseases, Endsleigh Gardens, 50 beds; King George's Sanatorium for Sailors at Bramshott, in Surrey, 80 beds; and a Convalescent Home at Cudham, in Kent, with 30 beds. In addition there is the London School of Tropical Medicine (attached to the Hospital in Endsleigh Gardens) of which Sir Perceval was also Chairman. In recognition of his services in connection with this last-named institution Sir Perceval Nairne was awarded the honour of knighthood in 1915.

The number of patients treated by the Seamen's Hospital Society when Sir Perceval joined the Board amounted to about 3,000 annually. Now over 18,000 receive care and treatment in the various establishments every year, while the income has risen from about £10,000 to nearly £60,000 and the expenditure from £8,000 to over £60,000 per annum.

Sir Perceval was a devoted Churchman and for over forty years was engaged in diocesan work. He was Honorary Secretary to the Rochester and Southwark Diocesan Conferences in succession for twenty-five years. For many years he was Vicar's Warden at St. Giles' Church, Camberwell, where he was baptised in 1841. It was in that church that the memorial service was held on December 14, 1921.

Up to recent years Sir Perceval was a very keen yachtsman and was the owner of the schooner, "Feronia." He was a prominent member of the

**The Oldest Hospital Chairman—(*cont.*).**

Freemasons' craft. In 1862 he was initiated in the Caveac Lodge at the "Crown and Sceptre Hotel," Greenwich, and his jubilee was celebrated at the special meeting of four lodges in 1912. In 1876 he was one of the founders of the Crichton Lodge, in which he was appointed Director of Ceremonies, and continued in that office up to his death. In 1892 he attained the office of Grand Deacon. He was also founder of several other lodges.

Many mourn the loss of Sir Perceval Nairne, but perhaps none will miss him more than the merchant seaman, for, in spite of his great age, he was unremitting in his efforts to advance the cause of the Hospital which cares for these men when ill. Only a short time before his death Sir Perceval wrote: "The Dreadnought stands as a record of voluntary effort for our Merchant Seamen. It is for this and the next gêneration to secure that the benefits of rapidly-advancing medical and surgical science are freely placed at the disposal of those whose courage and skill have stood the Empire in good stead in time of peril."

Mr. J. Cyril Nairne, Sir Perceval's nephew, is also a member of the Committee of Management of the Society, so the connection between the Charity and Sir Perceval's family will be maintained.

## Figures and Tables

**Figure f1:**